# Ex-Vivo Evaluation of X-Ray Horizontal Angle for Separating the Canals of Four-Canal First Mandibular Molars

**Published:** 2008-01-10

**Authors:** Jahangir Haghani, Maryam Raoof, Sadegh Pourahmadi

**Affiliations:** 1 *Department of Oral Radiology, Dental School, Kerman University of Medical sciences, Kerman, Iran*; 2 *Department of Endodontics, Dental School, Kerman University of Medical sciences, Kerman, Iran*; 3 *General Practitioner, Kerman, Iran*

**Keywords:** Endodontics, Molar, Radiology, Root Canal

## Abstract

**INTRODUCTION:** A variety of mesial and distal projections have been suggested for separating the canals in a multi-canaled root. But there is no general agreement on the best angulation for each tooth. The purpose of this study was to evaluate the X-ray horizontal angle for separating the canals of four-canal first mandibular molars.

**MATERIALS AND METHODS:** Forty four-canaled mandibular molars were selected. After preparation of coronal access cavities, files were inserted into the root canals and the specimens were radiographed at 10, 15, 20, 25 degrees mesial and distal horizontal angulations. Apices and canals were evaluated. Chi-square test was used for statistical analysis.

**RESULTS:** Although 10° and 15° mesial and distal angulations were best for the most obvious apices manifestation (P<0.001), it was found that 20° mesial angulation is significantly better than the other cone angulations (P<0.001) for separating the root canals.

**CONCLUSION:** 20° mesial angulation improved detection of both the canals and canal terminus visibility.

## INTRODUCTION

A key objective of successful nonsurgical endodontic treatment is effective chemo- mechanical debridement of the root canal systems (1). One factor in achieving this goal is establishment of precise working length (WL) (2).

Different techniques have been used for determining WL including: radiography, electronic devices, using paper point, tactile methods and patient reaction. None are totally accurate, so all the techniques must be used only as an adjunct to radiography (2).

Conventional intraoral radiography using silver halide film is a widely used and reliable clinical method of determining WL (3).

In a multi-canaled root the canals are superimposed one upon the other. Using special cone angulations, these structures can be moved apart to solve this problem (4).

However, as the horizontal angulation increases, the clarity of the radicular anatomy decreases (5). So having a clear image of the apex and therefore the WL determination would be more difficult.

A variety of mesial and distal projections have been suggested for effective “opening-up” the canals (5-7). But, there is no general agreement on the best angulation for each tooth. Therefore sometimes we need to obtain several radiographs that involve different cone angles with respect to the target tooth to have an acceptable image of all canals (8). This can result in economic and also biologic side effects.

The prevalence of four canals especially in mandibular first molar is noticeable and varies in different studies (9-12). On the other hand, this is the earliest permanent posterior tooth to erupt and seems to be the tooth that most often requires root canal treatment (13). So, suggesting an appropriate radiographic cone angulation may be helpful to better performing root canal therapy.

**Table 1:** Frequency and distribution of canals separation in mesial cone angulations

**Cone angulation (degree)**	**Separation of four canals (#40)**			
	Yes		No	
	Number	Percent	Number	Percent
10	0	0	40	100
15	15	37.5	25	62.5
20	33	82.5	7	17.5
25	20	50	20	50

The purpose of this ex-vivo study was to evaluate the effect of different x-ray tube angulations on defining the apices and separation of numerous root canals.

## MATERIALS AND METHODS

Included in this study were mandibular recently extracted molars with fully formed roots. The specimens with dilacerations, external root resorption and fracture had to be excluded. Teeth were washed immediately after extraction and stored in normal saline until the collection was completed. Thereafter, the teeth were properly washed under tap water and immersed in 5.25 % sodium hypochlorite for 30 min for the removal of organic debris on the surface. The specimens were radiographed from buccal view to assess canal morphology and those identified as having abnormal anatomy or calcified root canal systems were excluded.

Coronal access opening were prepared using tungsten carbide burs in a high speed handpiece. The canals were located using an endodontic explorer and 40 four-canal teeth were selected. The teeth immersed in 2.5% sodium hypochlorite solution for 24 hours to dissolve any pulp tissue. Root canal working length was set at 1 mm short of the apical foramen based on visual inspection of a size 10 stainless steel K-file penetrating beyond the apical foramen. The teeth were then mounted in self–cure acryl boxes. The teeth aspects were marked as M: mesial, B: buccal, D: distal and L: lingual. Canals were then prepared ( if necessary) by hand until a size 20 K-file (Maillefer, Dentsply, Swiss) bound at the working length. Files were inserted passively into the root canals. A horizontal goniometer which was able to show every 5 degrees was designed and a rectangular cardboard was installed on the radiographic tube head as a marker, these enabled precise variations in cone angulation. The specimens were adjusted on the scaled plate and the teeth were radiographed. The samples and dental x-ray unit were positioned to obtain a 0.8 cm focal spot–object distance. Conventional E–Speed intra-oral radiographic films (Kodak, Eastman Kodak, NY, USA) were exposed at 10,15,20,25 degrees mesial and distal horizontal angulations with the x-ray unit operating at 70 kvp, 8mA, for 0.32 s (Planmeca intra X-ray unit, Finland). Vertical angle was -5^°^ for all the specimens. Films were developed in an automatic processor (Velopxe, Extrax medien, UK) using champion developer and fixer solutions (champion photochemistry, Iran). A board- certified radiologist compared the images without magnification on a light viewing box (Shayanteb, Iran). He determined the apices and separation of the canals. No time limit was set for viewing and the observer was allowed a rest period whenever he felt fatigue. Chi-square test was performed for the statistical analysis using SPSS version 15.

**Table 2:** Frequency and distribution of canals separation in distal cone angulations

**Cone angulation (degree)**	**Separation of four canals (#40)**			
	Yes		No	
	Number	Percent	Number	Percent
10	0	0	40	100
15	10	25	30	75
20	16	40	24	60
25	19	47.5	21	52.5

## RESULTS

Results presented in Table 1 and Table 2 show that the mesial 20^°^ radiograph is significantly better than the other cone angulations for separating the root canals (P<0.001). There was no separation when 10^°^ mesial and distal angulations applied. The results concerning the apices defining with regard to different mesial and distal cone angulations are shown in Table 3, and Table 4, respectively. An increase in horizontal angle was found to be related to decrease of defining ability of the apices. Specifically, the angulations of 10^°^ and 15^°^ mesial and distal were best for the most obvious apices manifestation (P<0.001).

**Table 3:** Frequency and distribution of radiographic apices defining in mesial cone angulations

**Cone angulation (degree)**	**Separation of four canals (#40)**			
	Yes		No	
	Number	Percent	Number	Percent
10	40	100	0	0
15	40	100	0	0
20	36	90	4	10
25	11	27.5	29	27.5

## DISCUSSION

The prevalence of four canals in mandibular molars varies in different studies (8-11). The mesial root of the mandibular first molar usually has two canals. The prevalence of two canals in the distal root ranges from 11.2% to 43.3% (9-15). About the mandibular second molar, 21.4% to 56.9% of the mesial roots have been shown to have two canals (9,11,16,17).

This range is about 1/3% to 4% in distal root (9,11,16,17). Therefore, the prevalence of four root canals in mandibular molars is noticeable.

Careful assessment of pulp anatomy obtained from a diagnostic radiograph is very important to eradicate intra-radicular infection. Radiologic evaluation of the three dimensional configuration of the root and canal system is recommended in endodontics (18). We preferred conventional radiographs rather than digital radiographs because the dentists are more familiar with the former and the conventional radiography equipments are more available in many dental clinics. Moreover, the image quality of the conventional radiographs had been better than the older digital systems in some studies (19-21). The dental x-ray films used in this study (Kodak, E-speed, Eastman Kodak, Ny, USA) were chosen because of their routine use in dental clinics, while they have also higher resolution and better contrast (22).

It is more probable that an angled radiographic view will reveal additional information about the number of canals and apex visibility (23). According to this study, images taken at 20^°^ mesial angulation were significantly better than 10^°^, 15^°^ and 25^°^ angulations for detecting the number of canals. This finding was in conflict with Walton who has suggested the distal projection for mandibular molars (5). Ingle proposed 20^°^ to 30^°^ mesial shift to adequately reflect the morphologic characteristics of the mandibular molar root canal systems (6). In another in vitro study, Naoum *et al*. stated that conventional radiographs taken at a 0^°^ orientation were significantly better than 30^°^ images for detecting the number of visible canals (7).

**Table 4:** Frequency and distribution of radiographic apices defining in distal cone angulations

**Cone angulation (degree)**	**Separation of four canals (#40)**			
	Yes		No	
	Number	Percent	Number	Percent
10	40	100	0	0
15	40	100	0	0
20	32	80	8	20
25	6	15	34	85

In all 10^°^ mesial and distal images the canals were superimposed one upon the other and appeared as a single line. Moreover, when 10^°^ or 15^°^ mesial or distal radiographs were considered, it was obvious that the apices could be clearly visualized but the canals were not separated adequately. So, 20^°^ mesial angulation improved both detection of the canals and canal terminus visibility.

In the present study we attempted to follow steps that could be applied in vivo. It was difficult to achieve a precise horizontal angle. We solved this problem by designing a goniometer. The limitations of this ex-vitro study should be taken into account and indicate the need for further work.

## CONCLUSION

According to this study, it looks like that using 20° mesial angulation improves detection of both the canals and apices.
